# Vitamin D status and severity of COVID-19

**DOI:** 10.1038/s41598-022-21513-9

**Published:** 2022-11-17

**Authors:** Nete Munk Nielsen, Thor Grønborg Junker, Sanne Grundvad Boelt, Arieh S. Cohen, Kassandra L. Munger, Egon Stenager, Alberto Ascherio, Lasse Boding, Anders Hviid

**Affiliations:** 1 Department of Epidemiology Research, Statens Serum Institut, Copenhagen, Denmark; 2 Focused Research Unit in Neurology, Department of Neurology, Hospital of Southern Jutland, University of Southern Denmark, Aabenraa, Denmark; 3 Test Center Denmark, Statens Serum Institut, Copenhagen, Denmark; 4 Department of Nutrition, Harvard T.H. Chan School of Public Health, Boston, MA USA; 5 Multiple Sclerosis Clinic of Southern Jutland (Sønderborg, Kolding, Esbjerg), Department of Neurology, Hospital of Southern Jutland, University of Southern Denmark, Sønderborg, Denmark; 6 Department of Epidemiology, Harvard T.H. Chan School of Public Health, Boston, MA USA; 7 Channing Division of Network Medicine, Brigham and Women’s Hospital and Harvard Medical School, Boston, MA USA; 8 The Danish National Biobank, Statens Serum Institut, Copenhagen, Denmark; 9 Pharmacovigilance Research Centre, Department of Drug Design and Pharmacology, University of Copenhagen, Copenhagen, Denmark; 10 Section for Clinical Mass Spectrometry, Danish Center for Neonatal Screening, Department of Congenital Disorders, Statens Serum Institut, Copenhagen, Denmark; 11 iPSYCH, The Lundbeck Foundation Initiative for Integrative Psychiatric Research, Copenhagen, Denmark

**Keywords:** Diseases, Risk factors

## Abstract

We explored the association between COVID-19 severity and vitamin D status using information from Danish nation-wide health registers, the COVID-19 surveillance database and stored blood samples from the national biobank. 25-hydroxyvitamin D (25(OH)D) was measured using tandem mass spectroscopy. The association between 25(OH)D levels and COVID-19 severity, classified hierarchical as non-hospitalized, hospitalized but not admitted to an intensive care unit (ICU), admitted to ICU, and death, was evaluated by proportional odds ratios (POR) assuming proportionality between the four degrees of severity. Among 447 adults tested SARS-CoV-2 positive in the spring of 2020, low levels of 25(OH)D were associated with a higher risk of severe COVID-19. Thus, odds of experiencing more severe COVID-19 among individuals with insufficient (25 to < 50 nmol/L) and sufficient (≥ 50 nmol/L) 25(OH)D levels were approximately 50% of that among individuals with deficient levels (< 25 nmol/L) (POR = 0.49 (95% CI 0.25–0.94), POR = 0.51 (95% CI 0.27–0.96), respectively). Dividing sufficient vitamin D levels into 50 to < 75 nmol/L and ≥ 75 nmol/L revealed no additional beneficial effect of higher 25(OH)D levels. In this observational study, low levels of 25(OH)D were associated with a higher risk of severe COVID-19. A possible therapeutic role of vitamin D should be evaluated in well-designed interventional studies.

## Introduction

The majority of those infected with SARS-CoV-2 will have mild symptoms, but some will experience severe clinical symptoms necessitating hospitalization, and in worst cases, death^1,2^. It is still uncertain why some are more prone to develop a more severe outcome of COVID-19, but risk factors such as older age, male sex, obesity, non-white race and co-morbidities seem to be of importance^2^. Findings of a potential beneficial effect of vitamin D on the incidence and the severity of acute respiratory tract infections suggests that vitamin D deficiency also could be linked to the outcome of COVID-19 illness^3,4^.

Several studies have reported lower levels of vitamin D among individuals who test positive for SARS-CoV-2 as compared with those who test negative^5–7^. Other studies were not supportive of an association between vitamin D status and risk of SARS-CoV-2 infection^8–10^ but, in at least one case, the null result could be explained by the use of vitamin D levels measured several years in the past^10^. Clinical severity among hospitalized COVID-19 patients, evaluated according to the possible existence of unconsciousness, respiratory symptoms, organ dysfunction, need of invasive mechanical ventilation, or death have also been linked to lower vitamin D levels in some studies^11–15^, but not all^16–19^.

Despite the discrepancy in previous studies, findings from many of the systematic reviews and meta-analyses support the hypothesis that vitamin D deficiency is related to a higher SARS-CoV-2 infection risk and worse disease outcome^20–23^. However, clinical and methodological heterogeneity hamper comparison of the previous studies resulting in less reliable pooled risk estimates^22^. Especially selection bias, lack of adjustment for relevant confounders, timing of vitamin D measurements, and varying definitions of vitamin D deficiency are issues of great concern^5,6,12,14–16,18,22,24–26^.

Vitamin D is known to modulate the immune system, and a possible beneficial effect of vitamin D on the outcome of COVID-19 could be through the prevention of the overreaction of the immune system e.g. the cytokine storm characteristic of severe COVID-19^27,28^. Vitamin D may also play a role in the production of antimicrobial peptides in the respiratory epithelium, the maintenance of the integrity of the cellular junctions and the control of cellular proliferation and angiogenesis^27,28^. Furthermore, genetic variations in the vitamin D binding protein gene, specifically SNP in rs7041 locus, have been found to correlate with the prevalence of COVID-19 and mortality rates, suggesting that genetic factors may also have a role^29,30^.

COVID-19 hospitalizations continue, despite the availability of COVID-19 vaccines, and it is therefore urgent to determine whether vitamin D deficiency has a role in the severity of COVID-19 infection. In the present study, we assess the association between vitamin D status and COVID-19 severity among Danish COVID-19 patients using the Danish National Patient Registry, the Cancer Registry, the COVID-19 surveillance database, which contains information on all SARS-CoV-2 PCR tests carried out in Denmark, and blood samples stored in the National Biobank. Using these resources enable us to select the most suitable blood sample for every COVID-19 patient and adjust for relevant confounders.

## Methods

### The study cohort

Residual blood samples from clinical biochemistry departments at two major hospitals in the Capital Region of Copenhagen have routinely been sent to and stored by the Danish National Biobank (DNB) at Statens Serum Institut (SSI). Biological samples in the DNB are by the unique personal ID number assigned to every Danish citizen by the Civil Registration System (CRS)^31^ linked to the National Patient Registry (NPR)^32,33^ which enabled us to match stored blood samples in DNB drawn from individuals registered with a hospital contact due to COVID-19 up to the beginning of May 2020.

### Selection of blood samples for vitamin D measurements

A blood sample collected 1–30 days before registration of a COVID-19 diagnosis was preferred. If this was not possible, a blood sample drawn on the date of registration or 1–2 days after was selected, and if this could not be fulfilled, we selected the most recent blood sample collected up to 24 months before the COVID-19 registration. If none of the blood samples were drawn in this period, the person was excluded from the study.

### Measurements of vitamin D

For each participants’ sample, 25-hydroxyvitamin D (25(OH)D) was measured using 30 µl of blood plasma. We measured 25-hydroxyvitamin D_3,_ (25(OH)D3), which is the main circulating form of vitamin D, and the related ergosterol derived-form 25-hydroxyvitamin D_2_ (25(OH)D2). The assay was done at SSI and is highly sensitive using minimal sample cleanup to reduce sample loss during extraction, chemical derivatization to enhance 25(OH)D2 and 25(OH)D3 ionization, and liquid chromatography tandem mass spectroscopy coupled with multiple reaction monitoring^34^. Vitamin D level was calculated as the sum of 25(OH)D3 and 25(OH)D2.

### Information on SARS-CoV-2 test results

To confirm the diagnosis of COVID-19, the study cohort was linked to the SSI COVID-19 surveillance database, which includes results for every SARS-CoV-2 PCR test carried out in Denmark since February 2020. The database is based on information from the Departments of Clinical Microbiology and Test Center Denmark and is updated daily. In addition to test results, it also contains information on hospitalizations, admissions to ICU, and COVID-19 related death^35^.

### Severity of COVID-19

We defined onset of COVID-19 as date of a positive SARS-CoV-2 test and not as date of a hospital contact as we consider date of test to be closest to onset of first symptoms. A hospital contact related to COVID-19 was defined as a registration in the NPR with a diagnosis of COVID-19 (ICD-10 code; DB342A or DB972A) as primary or secondary diagnosis within 14 days after a positive test. However, if a hospitalized person with the above diagnostic codes tested positive within 2 days after admission, this hospitalization was also considered related to COVID-19. We defined four categories of increasing severity: (1) no hospital admission (incl. hospital contacts < 12 h), (2) hospitalized (≥ 12 h), but not admitted to ICU, (3) admitted to ICU, regardless of duration of hospital contact and (4) death within 30 days after a positive SARS-CoV-2 test.

### Potential confounders

As older age, male sex, chronic diseases, obesity, and non-white race have been associated with vitamin D deficiency^36,37^, as well as with an increased risk of severe COVID-19^2^, we consider those covariates as potential confounders. For each cohort member we therefore obtained detailed information on pre-existing diseases registered 5 years to 1 month prior to the positive SARS-CoV-2 test using information from the NPR^32,33^ and Cancer Registry^32,38^. The categories of pre-existing diseases were defined according to a modified version of the Charlson comorbidity index^39^ and we used the updated weights^40^ (Supplementary Table 1). Obesity was defined as any registration of clinical obesity in the NPR (ICD-10 codes: E65 or E66). We have no specific information on race/ethnicity, accordingly we used country of origin as proxy of race. Country of origin was defined using data from CPR on family relations and place of birth according to an algorithm developed by Statistics Denmark^41^.

### Statistical methods

25(OH)D levels were modelled in predefined categories, (< 25 nmol/L (deficient), 25 to < 50 nmol/L (insufficient), and ≥ 50 nmol/L (sufficient)) and as a continuous variable. We a posteriori divided the category of sufficient 25(OH)D levels into two levels; 50 to < 75 nmol/L and ≥ 75 nmol/L.

Assuming proportionality between the 4 categories of severity we used ordinal logistic regression to calculate proportional odds ratios (POR) to evaluate the possible association between severity of COVID-19 and vitamin D levels. We calculated profile likelihood based 95% confidence intervals to account for the non-normal distribution of the POR. We furthermore stratified the analyses according to age at SARS-CoV-2 test (< 65 and ≥ 65 years) and sex.

To adjust for variation in 25(OH)D due to seasonality during blood sample collection, we regressed the 25(OH)D levels on the periodic function *β*_*sine*_ sin(2ΠM_i_/12) + *β*_*cosinus*_ cos(2ΠM_i_ /12), where M_i_ is month of sample collection. The residuals from this model were added to the specific 25-hydroxyvitamin D means derived from the model to create an adjusted 25(OH)D as described by Munger et al.^42^. Besides adjusting for seasonality in vitamin D, we a priori included age, sex, Charlson comorbidity index, obesity and country of origin in the model.

### Supplementary analyses

We evaluated the validity of the proportional odds assumption, by estimating odds ratios of the pairwise comparisons of COVID-19 hospitalization, ICU and fatal COVID-19 vs no hospitalization, respectively.

### Sensitivity analyses

To further account for possible variations in vitamin D levels over calendar periods in 2018, 2019 and 2020 we restricted cohort members to those who had their blood samples drawn from the 1st of February 2020 to the 1st of May 2020. In a second sensitivity analysis we restricted cohort members to those who had a blood sample drawn 1–30 days before the positive SARS-CoV-2 test. We did this in order to minimize the risk of reverse causality i.e. that the COVID-19 illness would affect the person’s vitamin D level, and in order to eliminate the possibility of changes in vitamin D related behaviors after the blood sample was drawn e.g. beginning vitamin D supplementation.

### A posteriori analysis

Vitamin D levels appeared surprisingly high among the deceased COVID-19 cases. We speculate that this could reflect prescribed vitamin D supplementation to frail older persons. We therefore omitted death from the four categories of COVID-19 severity and estimated the odds of experiencing a more severe outcome of COVID-19 among individuals who survived the COVID-19.

### Ethics

This study was approved by the Danish National Committee on Health Research Ethics (H-20028195) and the Statens Serum Institut’s compliance department. We used already stored blood samples originally collected for other purposes. These blood samples were routinely sent to and stored by the National Biobank in Denmark. As the study was carried out (1) without active participation, (2) with no contact with any of the study participants, (3) without any health risks or other burdens for the persons whose data were used, and (4) including a large number of participants (originally approx. 2000), the Danish National Committee on Health Research Ethics granted us a dispensation from for the rule of obtaining consent in accordance with the Committee Act, section 16, subsection 3. All the analyses and the reporting of results have been done completely anonymously. The project group ensured that none of the blood sample owners had actively refused that their biological material could be used for research—this information is available in the Danish national “Tissue Use Register”.

## Results

**Figure 1:**
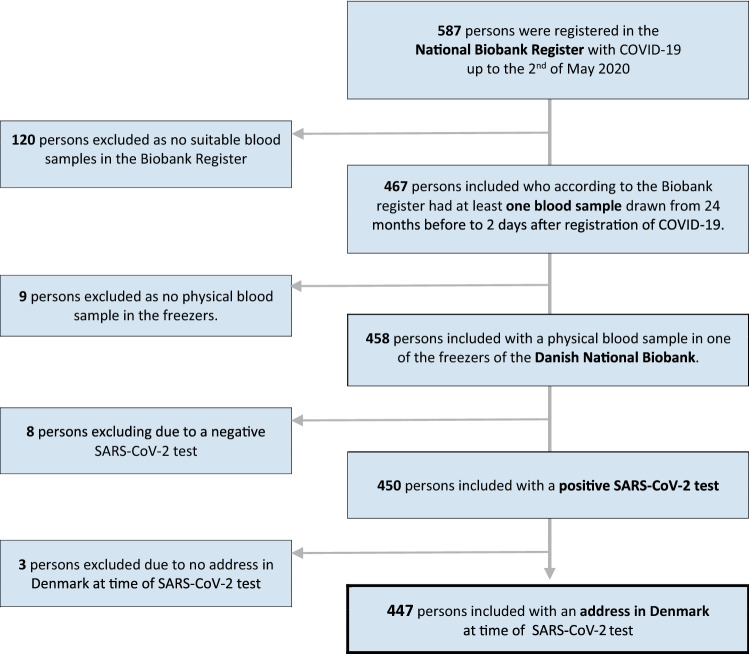
Flowchart showing the identification of the study participants.

Overall, 587 individuals with a least one blood sample in the DNB were registered with COVID-19 in the Danish Biobank Register. 120 persons did not have a blood sample drawn within the accepted time interval and 9 did not have a physical blood sample in the DNB, reducing the number of COVID-19 cases to 458. After linkage with the COVID-19 surveillance database 8 out the 458 did not have a positive SARS-CoV-2 test, and finally 3 persons were excluded as they had no address in Denmark at the time of the test (Fig. 1). The distribution of sex, mean age and vitamin D level of the 8 patients who did not have a positive SARS-CoV-2 test despite a COVID-19 registration did not differ from that among the 447 patients included in the study (data not shown).

Table 1 shows the characteristics of the 447 cohort members according to predefined categories of vitamin D levels. Overall, 11%, 30% and 59% of the cohort members had vitamin D deficiency, insufficiency or sufficiency, respectively (Table 1). Vitamin D level was statistically significantly associated with sex, age, country of origin and comorbidity, but not with obesity or time interval between date of blood sample drawn and SARS-CoV-2 test. Against expectations, we observed that 68% (145/212) of the cohort members ≥ 70 years had sufficient vitamin D levels versus 50% among individuals < 70 years (118/235), and that vitamin D sufficiency was more common among individuals with comorbidities than among individuals without comorbidities (68% (115/168) versus 53% (148/279), respectively).

**Table 1:** Summary of baseline characteristics of the study cohort.

	The total cohort N (%)	Vitamin D level			Homogeneity test
		< 25 nmol/L (Deficient) N (%)	25 to < 50 nmol/L (Insufficient) N (%)	≥ 50 nmol/L (Sufficient) N (%)	
**Total**	447 (100%)	49 (100%)	135 (100%)	263 (100%)	
**Sex**					
Male	219 (49.0)	31 (63.3)	78 (57.8)	110 (41.8)	
Female	228 (51.0)	18 (36.7)	57 (42.2)	153 (58.2)	P = 0.0011
**Age (years)**					
< 30	30 (6.7)	–	11 (8.2)	16 (6.1)	
30–39	31 (6.9)	6 (12.2)^a^	15 (11.1)	13 (4.9)	
40–49	43 (9.6)	8 (16.3)	19 (14.1)	16 (6.1)	
50–59	71 (15.9)	5 (10.2)	33 (24.4)	33 (12.6)	
60–69	60 (13.4)	5 (10.2)	15 (11.1)	40 (15.2)	
70–79	117 (26.2)	12 (24.5)	25 (18.5)	80 (30.4)	
≥ 80	95 (21.3)	13 (26.5)	17 (12.6)	65 (24.7)	P < 0.0001^b^
**Country-of-origin**					
Denmark	337 (75.4)	34 (69.4)	87 (64.4)	216 (82.1)	
Non-Western^c^	88 (19.7)	15 (30.6)^d^	43 (31.9)	33 (12.6)	
Western^e^	22 (4.9)	–	5 (3.7)	14 (5.3)	P < 0.0001
**Charlson comorbidity index**					
0	279 (62.4)	32 (65.3)	99 (73.3)	148 (56.3)	
1–2	140 (31.3)	17 (34.7)^f^	28 (20.7)	96 (36.5)	
≥ 3	28 (6.3)	–	8 (5.9)	19 (7.2)	P = 0.011
**Obesity**					
Yes	48 (10.7)	5 (10.2)	13 (9.6)	30 (11.4)	
No	399 (89.3)	44 (89.8)	122 (90.4)	233 (88.6)	P = 0.86
**Timing of blood collection in relation to SARS-CoV-2 test**					
1 year–2 years before test	104 (23.3)	–	37 (27.4)	63 (24.0)	
6 months–1 year before test	46 (10.3)	–	16 (11.9)	25 (9.5)	
1–6 months before test	27 (6.0)	11^g^ (22.4)	8 (5.9)	17 (6.5)	
1 day–1 month before test	79 (17.7)	8 (16.3)	20 (14.8)	51 (19.4)	
0 day–2 days after test	153 (34.2)	22 (44.9)	45 (33.3)	86 (32.7)	
More than 2 days after test	37 (8.3)	8 (16.3)	9 (6.7)	20 (7.6)	P = 0.17

^a^Due to few numbers the age groups < 30 and 30–39 years are grouped together in this table.

^b^Kruskal–Wallis test.

^c^Albania, Bosnia and Hercegovina, Belarus, Yugoslavia, Kosovo, Macedonia, Moldovia, Montenegro, Russia, Serbia, Soviet Union, Turkey, Ukraine, Africa, Asia, South and Central America, the Oceania (excl. Australia and New Zealand) and stateless people.

^d^Due to few numbers Non-Western and Western countries are grouped together in this table.

^e^EU, Andorra, Australia, Canada, Iceland, Liechtenstein, Monaco, New Zealand, Norway, San Marino, Switzerland, UK, US, The Vatican.

^f^Due to few number the categories of Charlson comorbidity index; 1–2 and ≥ 3 are grouped together.

^g^Due to few number the 3 periods covering the interval 1 month to 2 years are grouped together.

**Figure 2:**
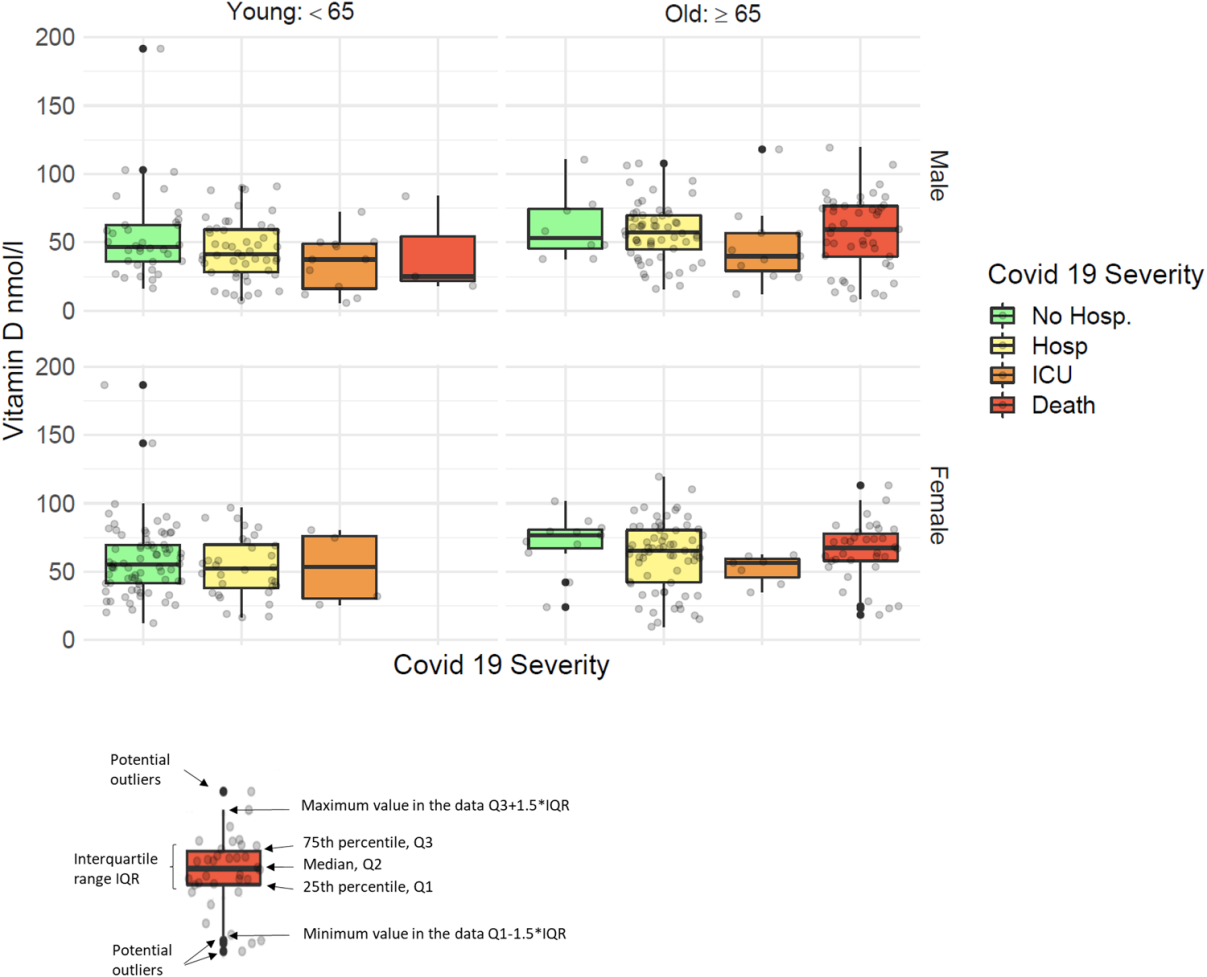
Boxplot with jitter showing the individual vitamin D levels (grey) according to degree of severity, age and sex.

Among the 447 cohort members, 126 (28%) were not hospitalized, 205 (46%) were hospitalized for COVID-19, but not admitted to an ICU, 34 (8%) were admitted to an ICU and 82 (18%) died from COVID-19. Figure 2 shows individual vitamin D levels in a boxplot according to COVID-19 severity, age (< 65 year and ≥ 65 years) and sex. Lower levels of vitamin D were associated with higher COVID-19 severity among males younger than 65 years of age but not among women of same age. Among the older men and women, the relationship between vitamin D levels and severity of COVID-19 resembled a U-formed association i.e. highest vitamin D levels were observed among those who were not hospitalized and among those who did not survive COVID-19 (Fig. 2).

In the main analysis, the odds of experiencing a more severe outcome of COVID-19 (progressing to the next severity category), among individuals with insufficient and sufficient levels of 25(OH)D were approximately 50% of that among individuals with deficient levels (POR = 0.49 (95% CI 0.25–0.94) and POR = 0.51 (95% CI 0.27–0.96), respectively) adjusted for age, sex, comorbidity, seasonality, obesity and country of origin (hereinafter referred to as aPOR). Treating vitamin D levels as a continuous variable, the odds of experiencing a more severe outcome of COVID-19 was reduced by 3% per 5 nmol/L increase in vitamin D level (aPOR = 0.97 (95% CI 0.93–1.01)). However, this estimate did not reach statistically significance. No beneficial effect of increasing levels of vitamin D was observed in the analysis dividing the category of vitamin sufficiency into 50 to < 75 nmol/L and ≥ 75 nmol/L (Table 2).

**Table 2:** Proportional Odds Ratios of developing more severe COVID-19 according to 25(OH)D levels, sex and age.

	Total			Age			
	N	POR (95% CI)	aPOR (95% CI)	< 65 years^b^		≥ 65 years	
				N	aPOR (95% CI)	N	aPOR (95% CI)
**Total**							
Predefined cutpoints of 25(OH)D levels							
< 25 nmol/L (Deficient)	49	1 (ref)	1 (ref)	23	1 (ref)	26	1 (ref)
25 to < 50 nmol/L (Insufficient)	135	0.36 (0.20–0.66)	0.49 (0.25–0.94)	86	0.43 (0.16–1.13)	49	0.49 (0.19–1.24)
≥ 50 nmol/L (Sufficient)	263	0.56 (0.32–0.98)	0.51 (0.27–0.96)	99	0.38 (0.14–1.05)	164	0.60 (0.26–1.36)
50 to < 75 nmol/L^a^	167	0.58 (0.32–1.03)	0.50 (0.26–0.96)	64	0.39 (0.13–1.10)	103	0.58 (0.24–1.36)
≥ 75 nmol/L^a^	96	0.54 (0.29–1.03)	0.53 (0.26–1.08)	35	0.38 (0.11–1.27)	61	0.64 (0.25–1.60)
Continuous levels of 25(OH)D							
Per 5 nmol/L	447	0.99 (0.96–1.02)	0.97 (0.93–1.01)	208	0.94 (0.88–1.01)	239	0.98 (0.93–1.04)
**Males**							
Predefined cutpoints of 25(OH)D levels							
< 25 nmol/L (Deficient)	31	1 (ref)	1 (ref)	17	1 (ref)	14	1 (ref)
25 to < 50 nmol/L (Insufficient)	78	0.32 (0.15–0.69)	0.35 (0.15–0.80)	46	0.44 (0.13–1.48)	32	0.26 (0.07–0.91)
≥ 50 nmol/L (Sufficient)	110	0.54 (0.26–1.12)	0.38 (0.17–0.87)	36	0.40 (0.11–1.44)	74	0.33 (0.09–1.08)
Continuous levels of 25(OH)D							
Per 5 nmol/L	219	0.99 (0.94–1.04)	0.97 (0.92–1.03)	99	0.92 (0.84–1.01)	120	1.00 (0.93–1.08)
**Females**							
Predefined cutpoints of 25(OH)D levels							
< 25 nmol/L (Deficient)	18	1 (ref)	1 (ref)	6	1 (ref)	12	1 (ref)
25 to < 50 nmol/L (Insufficient)	57	0.44 (0.17–1.15)	0.88 (0.28–2.80)	40	0.53 (0.09–3.42)	17	1.14 (0.24–5.34)
≥ 50 nmol/L (Sufficient)	153	0.82 (0.34–1.98)	0.84 (0.30–2.40)	63	0.37 (0.06–2.52)	90	1.20 (0.33–4.42)
Continuous levels of 25(OH)D							
Per 5 nmol/L	228	1.01 (0.96–1.06)	0.96 (0.90–1.02)	109	0.94 (0.83–1.04)	119	0.97 (0.89–1.05)

POR Proportional Odds Ratio, CI Confidence Interval, aPOR Proportional Odds Ratio adjusted for age, seasonal variation in vitamin D level, country of origin, Charlson comorbidity index, obesity and sex

^a^Reference group is still the group of individuals with deficient 25(OH)D levels.

^b^Due to few numbers the two highest comorbidity indices are grouped when adjusting.

Analyses stratified according to age (< 65 years, ≥ 65 years) and sex revealed estimates compatible with a higher severity of COVID-19 among vitamin D deficient males in all age groups and among women less than 65 years of age, whereas no such association was seen among women 65 years of age or older (Table 2). Numbers were few in the stratified analyses, as reflected by the rather wide confidence intervals surrounding the aPOR estimates, of which many did not reach statistically significance.

### Supplementary analyses

To evaluate the validity of the proportional odds assumption, we estimated the odds of progression in COVID-19 disease severity from non-hospitalized to hospitalized, non-hospitalized to ICU treatment and finally from non-hospitalized to death, according to vitamin D level. Overall, these results supported the validity of the main analysis based on proportional odds regression in that sufficient and insufficient levels protected against severe COVID-19 vs no hospitalization in pairwise comparisons (Supplementary Table 2).

### Sensitivity and a posteriori analyses

In the sensitivity analyses restricting cohort members to those with blood samples drawn in the spring of 2020 (n = 276) or to those with blood samples drawn within 1–30 days before positive test (n = 76), estimates remained supportive of a higher severity of COVID-19 among vitamin D deficient persons (Supplementary Table 3). Similar findings were observed in the a posteriori analyses in which we reduced the four severity levels to three levels by excluding cohort members who died within 30 days of a positive test (Supplementary Table 4).

## Discussion

We studied the association between vitamin D level and severity of COVID-19 among 447 individuals who tested positive within the first 3 months of the SARS-CoV-2 epidemic in Denmark. Overall, we found that individuals with vitamin D deficiency were at a higher risk of progressing to a more severe clinical outcome of COVID-19.

Most of the previous hospital-based studies observed an association between vitamin D deficiency and a more critical outcome of COVID-19. Vitamin D deficiency has been associated with elevated risk of oxygen support^43^, ICU admission^24^, invasive mechanical ventilation^11^, severe sepsis/septic shock^24^, lung damage^15^, hypoxia^13^, death^11,14,15^, higher level of the inflammatory marker CRP^13^ and severe/critical COVID-19 illness defined according to severity scales developed by WHO or CDC^13,14^. However, other studies observed no association between vitamin D deficiency and clinical outcome of COVID-19, such as ICU treatment^19^, requirement for mechanical ventilation^16,19^, mortality^16,19,25^, length of hospitalization^17,19^ and severe/critical COVID-19^17^ (WHO definitions).

Although some previous studious found no effect, a recent meta-analysis carried out by Dissanayake et al. including nearly 2 million adults and 76 studies concluded that vitamin D deficiency/insufficiency probably increases susceptibility to COVID-19 and severe COVID-19, although with a high risk of bias and heterogeneity, whereas association with mortality is less robust^22^. Future rigorous prospective studies, using a standardized definition of vitamin D deficiency, insufficiency and sufficiency will be essential.

Heterogeneity could be due to different definitions of vitamin D deficiency and of COVID-19 severity. We followed the definition given by the Danish National Board of Health claiming that vitamin D levels less than 25 nmol/L are considered deficient and levels ≥ 50 nmol/L as sufficient^44^, but in other studies vitamin D deficiency was defined as < 30 nmol/L^12^ or < 50 nmol/L^14,15^ and sufficient vitamin D levels as ≥ 75 nmol/L^17^. We did, however, a posteriori divide the highest level of vitamin D into 50 nmol/L to < 75 nmol/L and ≥ 75 nmol/L and found no evidence of an increasing beneficial effect of higher vitamin D levels on COVID-19 severity.

We defined severity of COVID-19 according to data routinely recorded in Danish Nationwide Health and administrative registers which facilitates a uniform and easy replicable rating of severity. In contrast to many of the other studies on COVID-19 we included non-hospitalized COVID-19 patients, which could have caused bias. However, to get a SARS-CoV-2 test in Denmark in the spring of 2020, a person had to be referred by a physician who had to assess whether a clinical evaluation at a hospital was required. If this was not the case the person was referred to a regional test center^45^. We therefore consider the majority of non-hospitalized in our study as representative of those referred to a hospital for clinical evaluation, but due to milder symptoms the assessing hospital physician did not considered inpatient admission necessary^45^. Certain discrepancies in the procedures of COVID-19 testing and registrations in the Danish health care system may however have existed at the beginning of the epidemic.

We are aware of the fact that by defining COVID-19 mortality as deceased within 30 days after a positive SARS-CoV-2 test, we may have included individuals who did not die due to COVID-19, but due to unrelated conditions. However, the proportion of deceased individuals infected with SARS-CoV-2 who died due to other causes than COVID-19 within 30 days after the test is considered to be low in Denmark until December 2021, the appearance of the omicron variant^46^.

A rather high proportion of the deceased COVID-19 patients in the present study had sufficient vitamin D levels i.e. ≥ 50 nmol/L. We speculate that this could be due to vitamin supplementation among the more vulnerable and elderly population. To evaluate the influence of confounding by indication we a posteriori repeated our analysis omitting study cohort members who did not survive COVID-19 and found compatible estimates.

Another important source to heterogeneity is timing of vitamin D measurements in relation to the test. Previous studies have tested vitamin D levels at time of admission, some during admission and few within the preceding 10 years^22^. Due to risk of reverse causality and possible vitamin D modifying conditions after the blood samples were drawn, e.g. use of vitamin D supplementation, we would have preferred to measure vitamin D in blood samples drawn 1–30 days before start of symptoms. This criterion could only be met for 18% of the COVID-19 patients in the present study. Although estimates lost statistical significance and confidence intervals were broad, we consider the results based on this smaller group of participants compatible with vitamin D deficiency being associated with an elevated risk of a more severe outcome of COVID-19.

We addressed the association between vitamin D status and COVID-19 severity among individuals infected in the spring of 2020 i.e. at the beginning of the epidemic. Several different variants of SARS-CoV-2 have appeared since then, with different patterns of severity and contagiousness, thus our findings may not be applicable to all SARS-CoV-2 infected individuals. Furthermore, COVID-19 vaccinations was initiated in Denmark in December 2020. The association between vitamin D status and severity of COVID-19 in the present study is therefore observed in a group of non-vaccinated persons.

We defined a COVID-19 related hospital contact as a registration in the NPR with a diagnosis of COVID-19 (ICD-10 code; DB342A or DB972A) as primary or secondary diagnosis within 14 days after a positive test or 2 days before a positive test. In situations where COVID-19 is recorded as a secondary diagnosis, we may have included some individuals hospitalized due to other causes than COVID-19, who was incidentally found SARS-CoV-2 positive. However, 87% of the COVID-19 hospitalizations in the present study were hospital contacts with a primary diagnosis of DB342A or DB972A. Another limitation could be false positive PCR test results. The specificity of the PCR test in Denmark has, however, been estimated as 99.85%, and accordingly we do not consider false positive PCR tests to have any influence on our results^47^.

Chronic health conditions have been shown to affect vitamin D levels and COVID-19 severity. We therefore adjusted for pre-existing diseases and obesity, however we cannot rule out the existence of residual confounding due to other health conditions not included in the Charlson comorbidity index.

## Conclusion

In this Danish observational study of 447 COVID-19 patients, we observed that deficient vitamin D levels were associated with an elevated risk of progressing to a more severe COVID-19 outcome.

The possible role of vitamin D supplementation in the treatment of COVID-19 has already been assessed in randomized controlled trials, but as pointed out in recent meta-analyses, large variations in vitamin D supplementation schemes in the hitherto published studies means that no definitive conclusion can be drawn on the therapeutic effect of vitamin D, until larger well-designed interventional studies addressing issues such as the appropriate dose, duration and mode of vitamin D administration have been completed^48–50^.

## Supplementary Information

Supplementary Tables.

## Data Availability

The data in the present study are considered person-sensitive, and cannot be shared due to data protection regulations.
