# Supplementary material for: Vitamin D status and severity of COVID-19
Source: Sci Rep. 2022 Nov 17;12:19823. doi: 10.1038/s41598-022-21513-9 (PMC9672358; doi:10.1038/s41598-022-21513-9)
Supplement: Supplementary file 1 — Supplementary Tables. [file 41598_2022_21513_MOESM1_ESM.pdf]

Supplementary table 1: ICD-10 coding algorithm for Charlson comorbidity index and weights (updated and classical)

Comorbidities	ICD10 codes	Weights[a]	
		Up-dated	Clas-sical
Myocardial infarction	I21,I22, I252	0	1
Congestive heart failure	I099, I110, I130, I132, I255, I420, I425-I429, I43, I50, P290	2	1
Peripheral vascular disease	I70, I71, I731, I738, I739, I771, I790, I792, K551, K558, K559, Z958, Z959	0	1
Cerebrovascular disease	I60-I69, G45, G46, H340	0	1
Dementia	F00-F03, F051, G30, G311	2	1
Chronic pulmonary disease	I278, I279, J40-J47, J60-J67, J684, J701, J703,	1	1
Rheumatic disease	M05, M06, M315, M32, M33, M34, M351, M353, M360	1	1
Peptic ulcer disease	K25-K28	0	1
Mild liver disease	B18, K701-K703, K709, K713-K715, K717, K73, K74, K760, K762-K764, K768, K769, Z944	2	1
Diabetes without chronic complications	E100, E101, E106, E108, E109, E110, E111, E116, E118, E119 E120, E121, E126, E128, E129, E130, E131, E136, E138, E139 E140, E141, E146, E148, E149	0	1
Diabetes with chronic complications	E102-E105, E107, E112-E115, E117, E122-E125, E127, E132-E135, E137, E142-E145, E147	1	2
Hemiplegia or paraplegia	G041, G114, G801, G802, G81, G82, G830-G834, G839	2	2
Renal disease	I120, I131, N032-N037, N052-N057, N18, N19, N250, Z490-Z492, Z940, Z992	1	2
Any malignancy with-out metastasis incl. lymphoma, leukemia	C00-C26, C30-C34, C37-C41, C43-C58, C60-C76, C81-C85, C88, C90-C97	2	2
Moderate or severe liver disease	I850, I859, I864, I982, K704, K711, K721, K729, K765, K766, K767, K72[b], I85[b]	4	3
Metastatic solid tumor	C77-C80	6	6
AIDS/HIV	B21, B22, B23[c], B24	4	6

[a]According to Ternavasio-de La vega et al. (Epidemiol. Infect. 2018)

[b]Quan et al. (Med Care 2005) did not include K72 (Hepatic failure, not elsewhere classified) and I85 (Oesophageal varices), but Thygesen et al. (BMC Medical Research Methodology 2011) did.

[c]Quan et al. did not include B23(Human immunodeficiency virus [HIV] disease resulting in other conditions) in there algorithm, but Thygesen et al. (BMC Medical Research Methodology 2011) did.

Supplementary table 2: Odds of COVID-19 disease progression from non-hospitalized to hospitalized, non-hospitalized to ICU treatment and non-hospitalized to death, according to vitamin D levels.

Vitamin D levels	1[a]	Odds ratio (OR) (95% CI)					
		2[a] versus 1[a]		3[a] versus 1[a]		4[a] versus 1[a]	
	N	N	aOR (95% CI)	N	aOR (95% CI)	N	aOR (95% CI)
**Predefined cut-points of 25(OH)D**							
<25 nmol/L (Deficient)	7	23	1 (ref)	6	1 (ref)	13	1 (ref)
25 to <50 nmol/L (Insufficient)	48	60	0.50 (0.16-1.42)	14	0.42 (0.09-1.96)	13	0.44 (0.05-3.47)
≥50 nml/L (Sufficient)	71	122	0.58 (0.18-1.65)	14	0.25 (0.05-1.25)	56	0.62 (0.08-3.87)
50 to <75nmol/L[b]	41	83	0.64 (0.20-1.91)	<14	0.33 (0.06-1.75)	31	0.62 (0.08-4.33)
≥75nmol/L[b]	30	39	0.47 (0.13-1.53)	<14	0.09 (0.01-0.75)	25	0.62 (0.07-4.71)
**Continuous levels of 25(OH)D**							
Per 5 nmol/L	126	205	0.97 (0.91-1.03)	34	0.85 (0.74-0.96)	82	0.97 (0.85-1.09)

aOR=Odds Ratio adjusted for age, seasonal variation in vitamin D level, country of origin, Charlson comorbidity index, obesity and sex. CI= Confidence Interval

[a] Severity of COVID-19, 1=No hospital contacts (incl. hospital contacts < 12 hours), 2=Hospitalized (≥ 12 hours), but, not admitted to an intensive care unit (ICU), 3=Admitted to ICU, regardless of duration of hospitalization and, 4=Death.

[b] Reference group is still the group of individuals with deficient levels.

Supplementary table 3: Sensitivity analyses

**Vitamin D levels**	**Proportional odds Ratio (POR) (95% CI)**		
	N	POR	aPOR (95% CI)
**SENSITIVITY ANALYSIS 1**			
Blood samples 1st of February to 1st May 2020			
**Predefined cut-points of 25(OH)D categories**			
<25 nmol/L (Deficient)	39	1 (ref)	1 (ref)
25 to <50 nmol/L (Insufficient)	77	0.76 (0.37-1.58)	0.78 (0.36-1.66)
≥50 nml/L (Sufficient)	160	0.81 (0.42-1.59)	0.51 (0.24-1.04)
50 to <75nmol/L[a]	105	0.80 (0.40-1.62)	0.48 (0.22-1.02)
≥75nmol/L[a]	55	0.83 (0.37-1.86)	0.57 (0.24-1.33)
**Continuous levels of 25(OH)D**			
Per 5 nmol/L	276	0.99 (0.95-1.04)	0.96 (0.91-1.01)
**SENSITIVITY ANALYSIS 2**			
Blood sample collected 1-30 days prior to positive test.			
**Predefined cut-points of 25(OH)D categories**			
<25 nmol/L (Deficient)	8	1 (ref)	1 (ref)
25 to <50 nmol/L (Insufficient)	20	0.80 (0.17-3.57)	0.59 (0.10-3.12)
≥50 nml/L (Sufficient)	51	0.61 (0.15-2.32)	0.22 (0.04-1.08)
50 to <75nmol/L[a]	32	0.56 (0.13-2.27)	0.18 (0.03-0.94)
≥75nmol/L[a]	19	0.70 (0.15-3.20)	0.34 (0.05-2.08)
**Continuous levels of 25(OH)D**			
Per 5 nmol/L	79	0.98 (0.89-1.07)	0.92 (0.82-1.02)

POR=Proportional odds Ratio, CI= Confidence Interval, aPOR=POR adjusted for age, country of origin, Charlson comorbidity index, obesity and sex. CI=Confidence Intervals.

[a]Reference group is still the group of individuals with deficient levels.

Supplementary table 4: Proportional Odds Ratios of developing more severe COVID-19 according to 25(OH)D levels and age, excluding death from the severity scale.

	Total			Age			
				<65 years		≥65 years	
	N	POR (95% CI)	aPOR (95% CI)	N	aPOR (95% CI)	N	aPOR (95% CI)
**TOTAL**							
**Predefined cutpoints of 25(OH)D levels**							
<25 nmol/L (Deficient)	36	1 (ref)	1 (ref)	22	1 (ref)	14	1 (ref)
25 to <50 nmol/L (Insufficient)	122	0.42 (0.20-0.90)	0.59 (0.26-1.32)	85	0.41 (0.15-1.09)	37	0.98 (0.21-4.67)
≥50 nmol/L (Sufficient)	207	0.45 (0.22-0.92)	0.46 (0.21-1.01)	98	0.32 (0.12-0.89)	109	0.62 (0.16-2.54)
50 to <75nmol/L[a]	74	0.55 (0.26-1.14)	0.54 (0.24-1.21)	64	0.34 (0.12-0.96)	72	0.83 (0.20-3.64)
≥75nmol/L[a]	36	0.31 (0.14-0.70)	0.32 (0.13-0.80)	34	0.25 (0.07-0.84)	37	0.32 (0.07-1.58)
**Continuous levels of 25(OH)D**							
Per 5 nmol/L	365	0.95 (0.91-0.99)	0.93 (0.89-0.98)	205	0.92 (0.86-0.99)	160	0.91 (0.84-1.00)

POR=Proportional odds Ratio, CI= Confidence Interval. aPOR=POR adjusted for age, seasonal variation in vitamin D level, country of origin, Charlson comorbidity index, obesity and sex.
[a]Reference group is still the group of individuals with deficient levels.
